# Molecular Docking, Synthesis, and Tyrosinase Inhibition Activity of Acetophenone Amide: Potential Inhibitor of Melanogenesis

**DOI:** 10.1155/2022/1040693

**Published:** 2022-01-11

**Authors:** Yasir Nazir, Hummera Rafique, Sadia Roshan, Shazia Shamas, Zaman Ashraf, Muhammad Rafiq, Tehreem Tahir, Zia-Ur-Rahman Qureshi, Alvina Aslam, Muhammad Hassham Hassan Bin Asad

**Affiliations:** ^1^Department of Chemistry, Allama Iqbal Open University, Islamabad, Pakistan; ^2^Faculty of Sciences, Department of Chemistry, University of Sialkot, Sialkot, Pakistan; ^3^Department of Chemistry, University of Gujrat, Gujrat 50700, Pakistan; ^4^Department of Zoology, University of Gujrat, Gujrat 50700, Pakistan; ^5^Department of Physiology and Biochemistry, Faculty of Bio-Sciences, Cholistan University of Veterinary and Animal Sciences, Bahawalpur 63100, Pakistan; ^6^Institute of Biochemistry, Biotechnology and Bioinformatics, Faculty of Science, The Islamia University of Bahawalpur, Bahawalpur 63100, Pakistan; ^7^Department of Pharmacy, SBK Women University, Quetta, Pakistan; ^8^Institute of Molecular Biology and Biotechnology (IMBB), University of Lahore, Punjab, Pakistan; ^9^Department of Pharmacy, COMSATS University Islamabad, Abbottabad Campus, 22060, Pakistan

## Abstract

Tyrosinase and its related proteins are responsible for pigmentation disorders, and inhibiting tyrosinase is an established strategy to treat hyperpigmentation. The carbonyl scaffolds can be effective inhibitors of tyrosinase activity, and the fact that both benzoic and cinnamic acids are safe natural substances with such a scaffolded structure, it was speculated that hydroxyl-substituted benzoic and cinnamic acid derivatives may exhibit potent tyrosinase inhibitory activity. These moieties were incorporated into new chemotypes that displayed in vitro inhibitory effect against mushroom tyrosinase with a view to explore antimelanogenic ingredients. The most active compound, 2-((3-acetylphenyl)amino)-2-oxoethyl(E)-3-(2,4-dihydroxyphenyl)acrylate (5c), inhibited mushroom tyrosinase with an IC_50_ of 0.0020 ± 0.0002 *μ*M, while 2-((3-acetylphenyl)amino)-2-oxoethyl 2,4-dihydroxybenzoate (3c) had an IC_50_ of 27.35 ± 3.6 *μ*M in comparison to the positive control arbutin and kojic acid with a tyrosinase inhibitory activity of IC_50_ of 191.17 ± 5.5 *μ*M and IC_50_ of 16.69 ± 2.8 *μ*M, respectively. Analysis of enzyme kinetics revealed that 5c is a competitive and reversible inhibitor with dissociation constant (Ki) value 0.0072 *μ*M. In silico docking studies with mushroom tyrosinase (PDB ID 2Y9X) predicted possible binding modes in the enzymatic pocket for these compounds. The orthohydroxyl of the cinnamic acid moiety of 5c is predicted to form hydrogen bond with the active site side chain carbonyl of Asn 260 (2.16 Å) closer to the catalytic site Cu ions. The acetyl carbonyl is picking up another hydrogen bond with Asn 81 (1.90 Å). The inhibitor 5c passed the panassay interference (PAINS) alerts. This study presents the potential of hydroxyl-substituted benzoic and cinnamic acids and could be beneficial for various cosmetic formulations.

## 1. Introduction

A binuclear copper containing metalloenzyme tyrosinase has been detected in various species including fungi, bacteria, plants, and animals [1]. It is primarily involved in catalytic oxidation of its natural substrates L-tyrosine and L-DOPA (L-3,4-dihydroxyphenylalanine) to dopaquinone in a rate-limiting step during melanin biosynthesis which is associated with the reduction of oxygen (O_2_) into water [2, 3]. The dopaquinone synthesized *in vivo* in specialized organelles called melanosomes undergo further oxidation and several nonenzymatic polymeric reactions to form brown/black eumelanin and by reacting with cysteine and thiols to yellow/red pheomelanin [4–6]. Melanin is mainly responsible for skin, hair, and eye color and protects DNA degradation by ultraviolet (UV) radiation by acting as a photoprotectant in living organisms. The UV rays initiate melanogenesis for protecting skin cells from detrimental effects [7]. However, an abnormal production and accumulation of melanin in the dermal layer could cause several dermatological disorders including melasma, freckle, lentigo, and Riehl melanosis [8, 9]. To date, numerous approaches including the tyrosinase inhibition, melanocyte to keratinocyte melanin transfer suppression, and cell signaling intervention for melanogenesis have been attempted to combat the increased amount of melanin [10–14]. Many natural and synthetic antimelanogenic substances with promising characteristics in both *in vivo* and *in vitro* have been reported [15, 16], but the majority is associated with various side effects involving an in sufficient potency, permanent depigmentation and dermatitis [17, 18], thyroid cancer [19], nephrotoxicity [20], genotoxicity [21], and toxicity to melanocytic cells [22]. Therefore, the search for novel inhibitors of melanogenesis is of valuable concern to both medicinal and cosmeceutical industries. The tyrosinase inhibitory activity of acetophenone moiety has only been rarely reported [23]. Nazir et al. and Rafiq et al. reported that hydroxyl-substituted benzoic/cinnamic acid analogues exhibited potent tyrosinase inhibition [24, 25].

Over the past several years, our laboratory has reported various phenolic derivatives including tyramine, thymol, vanillin, carvacrol, and coumarin analogues which structurally mimic the natural substrates of the enzyme tyrosinase stronger than the L-tyrosine and L-DOPA [26–30]. We concluded from these investigations that substitution pattern of phenolic hydroxyls is directly related to degree of tyrosinase inhibition. Keeping in view these findings, the amide-based hydroxyl substituted benzoic/cinnamic acid analogues were synthesized to explore their tyrosinase inhibitory activity both in silico and *in vitro* so that more potent inhibitors are engineered. The synthesized benzoic 3(a–e) and cinnamic acids 5(a–e) derivatives passed the panassay interference screening except 3d [31]. The enzyme inhibitory kinetics and reversibility of the protein inhibitor complex were determined using Lineweaver-Burk and Dixon plots. The computational molecular docking was also performed against the crystal structure of target mushroom tyrosinase (PDB ID: 2Y9X) to predict the role of various amino acid residues in ligand-protein complex formation for further insights into biological properties.

## 2. Materials and Methods

### 2.1. General

Dry dichloromethane (DCM) was dried following the standard method. The IR-spectra (cm^−1^) were taken with a FT-IR spectrophotometer (Perkin Elmer, USA) using attenuated total reflection sampling technique (ATR). NMR spectra were taken using a Bruker spectrometer (^1^H, 400 MHz, ^13^C, 100 MHz, DMSO-*d_6_*), and chemical shifts (*δ*) are reported in parts per million (ppm). The Flash elemental analyzer connected with thermal conductivity detector (Flash-TCD 2000 Series, USA) was used to perform elemental analysis (C, H) and reported uncorrected with ±0.3%. The melting points determined by Digimelt MPA 160 (USA) are reported uncorrected. Silica gel thin layer column chromatography-TLC was used to check the purity of title derivatives (petroleum spirit : ethyl acetate, 2 : 1). All other reagents such as the enzyme mushroom tyrosinase (Mtyr, EC 1.14.18.1), L-DOPA, and 1-(3-acetylphenyl)ethan-1-one were analytical grade (Sigma Aldrich, USA) and are used without further purification.

### 2.2. Chemistry

#### 2.2.1. General Procedure for the Synthesis of N-(3-Acetylphenyl)Benzoates 3(a–e) and Cinnamates 5(a–e)

**Scheme 1:**
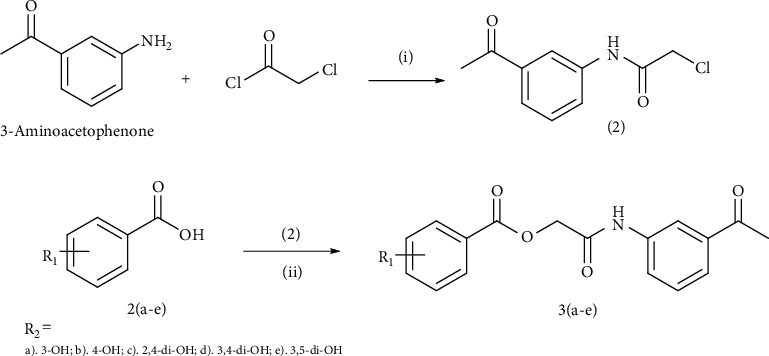
Synthetic pathway for title derivatives 3(a–e); reaction conditions and reagents; (i) dichloromethane (CH_2_Cl_2_)/triethylamine (C_2_H_5_)_3_N, 0-5°C, 5 hours stirring (ii) dimethylformamide (DMF)/(C_2_H_5_)_3_N/KI, stirring for 24 hours at room temperature.

**Scheme 2:**
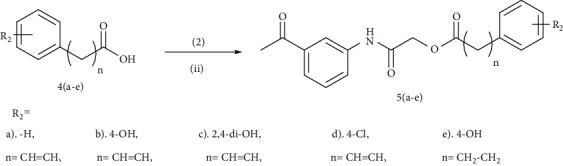
Synthetic pathway for title compounds 5(a–e); reaction conditions and reagents; (ii) dimethylformamide (DMF)/(C_2_H_5_)_3_N/KI, stirring for 24 hours at room temperature.

The intermediate *N*-(3-acetylphenyl)-2-chloroacetamide (2) synthesized by following Sidhu et al., with little modifications [32], was condensed with various hydroxyl-substituted benzoic acids 2(a–e) and cinnamic acids 4(a–e) (0.01 mol) with equimolar triethylamine and potassium iodide (KI) in dimethylformamide (DMF) (25 mL) under nitrogen at 25°C and stirred overnight. The reaction mixture was then concentrated using rotary evaporator and extracted using ethyl acetate (3 × 25 mL). Finally, the combined ethyl acetate layer was treated with HCl (5%) and brine followed by drying over magnesium sulphate MgSO_4_, filtered and concentrated by rotary evaporator to get the crude title products 3(a–e) and 5(a–e) (Schemes 1 and 2) and further purified by normal phase column chromatography (petroleum spirit : ethyl acetate, 3 : 1).

#### 2.2.2. The Spectroscopic Characterization of Synthesized Compounds 3(a–e) and 5(a–e)

The ^1^H-NMR of final compounds 3(a–e) and 5(a–e) showed an overlap of peaks in the alkyl region for homologues of this series. The singlet of terminal ketonic methyl proton of peripheral phenyl ring occupies the most upfield region of the spectrum encompassing the range of 2.54-2.56 ppm. In ^13^C NMR, the carbon of this terminal ketonic methyl group resonates between chemical shifts 26.73 and 26.80 ppm. The most shielded protons in the peripheral chain were the methylene (-CH_2_) proton which are directly linked to ester groups. This methylene group has a chemical shift value of 4.67-4.94 ppm while the carbon shift of this group in ^13^C NMR was found between 61.92 and 63.13 ppm. The acetyl phenyl amide lies in the range 165.69-168.28 ppm whereas carboxylic ester was found at 165.37-172.06 ppm.

*(1) (3a) 2-(3-Acetylphenylamino)-2-Oxoethyl Hydroxybenzoate*. Solid; Mp: 138-140°C; R_f_: 0.57, FT-IR (cm^−1^): 3421, 3245, 2901, 2865, 1725 (CO ester), 1632 (CO amide), 1589 (C=C aromatic): ^1^H-NMR; *δ* =10.52 (1H, s, NH), 9.91 (1H, s, OH), 8.20 (s, 1H), 7.85 (d, *J* =8.1 Hz, 1H), 7.68 (d, *J* =7.4 Hz, 1H), 7.47 (dd, *J* =7.2, 8.1 Hz, 2H) 7.35 (t, *J* =7.7 Hz, 1H), 7.08 (d, *J* =8.5 Hz, 1H), 4.93 (2H, s, CH_2_), 2.55 (3H, s, CH_3_); ^13^C-NMR; *δ* =197.6 (CO, ketone), 165.8 (CO, amide), 165.5 (CO, ester), 157.6, 138.9, 137.4, 130.4, 129.9, 123.9, 123.7, 120.7, 120.1, 118.5, 118.4, 115.9, 63.13 (CH_2_), 26.8 (CH_3_) (Figure S1); Molecular formula: C_17_H_15_NO_5_; Elemental Analysis: Calculated: (C, H) 65.17, 4.83; Found: (C, H) 65.19, 4.85.

*(2) (3b) 2-(3-Acetylphenylamino)-2-Oxoethyl 4-Hydroxybenzoate*. Solid; Mp: 146-148°C; R_f_: 0.56, FT-IR (cm^−1^): 3456, 3266, 2943, 2866, 1721 (CO ester), 1640 (CO amide), 1601 (C=C aromatic); ^1^H-NMR; *δ* =10.51 (1H, s, NH), 10.42 (1H, s, OH), 8.18 (s, 1H), 7.89 (d, *J* =8.8 Hz, 2H), 7.83 (d, *J* =6.8 Hz, 1H), 7.68 (d, *J* =8.1 Hz, 1H), 7.48 (t, *J* =6.1 Hz, 1H), 6.89 (d, *J* =8.7 Hz, 2H), 4.88 (2H, s, CH_2_), 2.55 (3H, s, CH_3_); ^13^C-NMR; *δ* =197.6 (CO, ketone), 165.8 (CO, amide), 165.48, 157.58 (CO, ester), 138.91, 137.38, 130.4, 129.89, 123.75, 120.68, 120.13, 118.45, 115.9, 63.13, (CH_2_), 26.75 (CH_3_) (Figure S2); Molecular formula: C_17_H_15_NO_5_; Elemental Analysis: Calculated: (C, H) 65.17, 4.83; Found: (C, H) 65.15, 4.81.

*(3) (3c) 2-(3-Acetylphenylamino)-2-Oxoethyl 2,4-Dihydroxybenzoate*. Solid; Mp: 210-212°C; R_f_: 0.51, FT-IR (cm^−1^): 3464, 3245, 2934, 2867, 1723 (CO ester), 1649 (CO amide), 1594 (C=C aromatic): ^1^H-NMR; *δ* =10.53 (1H, s, OH), 10.47 (1H, s, OH), 10.44 (1H, s, NH), 8.16 (s, 1H), 7.86-7.69 (m, 3H), 7.49 (d, *J* =7.7 Hz, 1H), 6.42 (d, *J* =8.7 Hz, 1H), 6.34 (s, 1 H), 4.94 (2H, s, CH_2_), 2.56 (3H, s, CH_3_); ^13^C-NMR; *δ* =197.57 (CO, ketone), 168.28 (CO, amide), 165.65 (CO, ester), 164.49, 162.7, 138.79, 137.39, 132.13, 129.33, 123.76, 118.44, 118.15, 108.48, 103.86, 102.58, 62.85 (CH_2_), 26.74 (CH_3_) (Figure S3); Molecular formula: C_17_H_15_NO_6_; Elemental Analysis: Calculated: (C, H) 62.00, 4.59; Found: (C, H) 62.02, 4.61.

*(4) (3d) 2-(3-Acetylphenylamino)-2-Oxoethyl 3,4-Dihydroxybenzoate*. Solid; Mp: 188-190°C; R_f_: 0.49, FT-IR (cm^−1^): 3423, 3234, 2923, 2867, 1727 (CO, ester), 1637 (CO amide), 1596 C=C aromatic); ^1^H-NMR; *δ* =10.41 (1H, s, NH), 8.17 (d*, J* =8.7 Hz), 7.84 (t, *J* =6.3 Hz, 1H), 7.69 (t, *J* =8.1 Hz, 1H), 7.44 (s, 2H), 7.40 (dd, *J* =2.0, 8.1 Hz, 1H), 7.44 (s, 2H), 6.85 (d, *J* =8.1 Hz, 1H), 4.86 (2H, s, CH_2_), 4.28 (2H, s, OH), 2.56 (3H, s, CH_3_); ^13^C-NMR; *δ* =197.60 (CO, ketone), 166.10 (CO, amide), 165.37 (CO, ester), 150.79, 145.14, 138.93, 137.39, 129.37, 123.85, 122.26, 119.98, 118.53, 115.39, 62.77 (CH_2_), 26.375 (CH_3_) (Figure S4); Molecular formula: C_17_H_15_NO_6_; Elemental Analysis: Calculated: (C, H) 62.00, 4.59; Found: (C, H) 61.99, 4.57.

*(5) (3e) 2-(3-Acetylphenylamino)-2-Oxoethyl Dihydroxybenzoate*. Solid; Mp: 210-221°C; R_f_: 0.46, FT-IR (cm^−1^): 3435, 3269, 2938, 2849, 1732 (CO ester), 1654 (CO amide), 1599 (C=C aromatic); ^1^H-NMR; *δ* =10.43 (1H, s, NH), 9.70 (2H, s, OH), 8.18 (s, 1H), 7.84 (d, *J* =8.0 Hz, 1H), 7.68 (d, *J* =8.0 Hz, 1H), 7.48 (t, *J* =7.6 Hz, 1H), 6.92 (s, 1H), 6.49 (s, 1H), 4.90 (2H, s, CH_2_), 2.56 (3H, s, CH_3_); ^13^C-NMR; *δ* =197.63 (CO, ketone), 165.86 (CO, amide), 165.57 (CO, ester), 158.65, 138.92, 137.41, 130.88, 129.35, 123.97, 123.89, 123.78, 123.68, 118.45, 107.57, 107.47, 63.12 (CH_2_), 26.77 (CH_3_) (Figure S5): Molecular formula: C_17_H_15_NO_6_; Elemental Analysis: Calculated: (C, H) 6200, 4.59: Found: (C, H) 61.98, 4.57.

*(6) (5a) 2-(3-Acetylphenylamino)-2-Oxoethyl Cinnamate*. Solid; Mp: 159-161°C; R_f_: 0.58, FT-IR (cm^−1^): 3234, 2935, 2865, 1731 (CO ester), 1641 (CO amide), 1598 (C=C aromatic); ^1^H-NMR; *δ* =9.94 (1H, s, NH), 8.33 (s, 1H), 7.98 (d, *J* =7.6 Hz, 1H), 7.87 (d, *J* =7.6 Hz, 1H), 7.77-7.65 (m, 3H), 7.50-7.45 (m, 3H), 4.84 (2H, s, CH_2_), 2.55 (3H, s, CH_3_); ^13^C-NMR; *δ* =197.59 (CO, ester), 145.38, 138.94, 137.36, 133.94, 130.69, 128.99, 128.49, 124.13, 123.32, 119.1, 118.5,17.46, 61.92 (CH_2_), 26.74 (CH_3_) (Figure S6); Molecular formula: C_19_H_17_NO_4_; Elemental Analysis: Calculated: (C, H) 70.58, 5.30; Found: (C, H) 70.60, 5.32.

*(7) (5b) 2-(3-Acetylphenylamino)-2-Oxoethyl (E)-3-(4-Hydroxyphenyl) Acrylate*. Solid; Mp: 227-229°C; R_f_: 0.49, FT-IR (cm^−1^): 3474, 3255, 2943, 2862, 1730 (CO ester, 1644 (CO amide), 1597 (C=C aromatic); ^1^H-NMR; *δ* =10.39 (1H, s, NH), 10.09 (1H, s, OH), 8.19 (s, 1H), 7.86 (d, *J* =8.2 Hz, 1H), 7.65 (d, *J* =8.0, 16.0 Hz, 3H), 7.59 (d, *J* =8.0 Hz, 1H), 7.47 (d, *J* =3.2, 8.2 Hz, 1H). 6.83 (d, *J* =8.0 Hz, 2H), 6.51 (d, *J* =16.0 Hz, 1H), 4.80 (2H, s, CH_2_), 2.56 (3H, s, CH_3_); ^13^C-NMR; *δ* =197.59 (CO, ketone), 166.21 (CO, amide), 166.07 (CO, ester), 160.09, 145.64, 138.90, 137.36, 129.28, 125.03, 123.82, 123.62, 118.52, 115.87, 113.48, 62.48, 62.50 (CH_2_), 26.73 (CH_3_) (Figure S7); Molecular formula: C_19_H_17_NO_5_; Elemental Analysis: Calculated: (C, H) 67.25, 5.05; Found: (C, H) 67.23, 5.03.

*(8) (5c) 2-(3-Acetylphenylamino)-2-Oxoethyl (E)-3-(2,4-Dihydroxyphenyl) Acrylate*. Solid; Mp: 233-235°C; R_f_: 0.46, FT-IR (cm^−1^): 3459, 3249, 2931, 2861, 1739 (CO ester), 1639 (CO amide), 1591 (C=C aromatic); ^1^H-NMR; *δ* =10.34 (1H, s, OH), 10.22 (1H, s, OH), 9.93 (1H, s, NH), 8.18 (s, 1H), 7.85 (d, *J* =8.0 Hz, 1H), 7.80 (d, *J* =8.4 Hz, 1H), 7.74 (d, *J* =16.0 Hz, 1H), 7.68 (d, *J* =7.7 Hz, 1H), 7.49 (d, d, *J* =8.0, 7.6 Hz, 2H), 6.79 (d, *J* =16.0 Hz, 2H), 4.76 (2H, s, CH_2_), 2.50 (3H, s, CH_3_): ^13^C-NMR; *δ* =197.61 (CO, ketone), 166.81 (CO, amide), 166.24 (CO, ester), 161.25, 158.78, 141.65, 138.93, 137.37, 130.69, 129.28, 123.81, 123.60, 119.13, 118.51, 112.57, 112.19, 107.94, 62.36 (CH_2_), 26.75 (CH_3_) (Figure S8); Molecular formula: C_19_H_17_NO_6_; Elemental Analysis: Calculated: (C, H) 64.22, 4.82; Found: (C, H) 64.23, 4.84.

*(9) (5d) 2-(3-Acetylphenylamino)-2-Oxoethyl (E) -3-(4-Chlorophenyl) Acrylate*. Solid; Mp: 182-184°C: R_f_: 0.53, FT-IR (cm^-1)^: 3267, 2932, 2865, 1726, 1645 (CO amide), 1598 (C=C aromatic); ^1^H-NMR; *δ* =10.44 (1H, s, NH), 8.18 (s, 1H), 7.85 (d, *J* =8.1 Hz, 1H),7.80 (d, *J* =8.3 Hz, 1H), 7.74 (d, *J* =16.0 Hz, 1H), 7.68 (d, *J* =7.7 Hz, 1H), 7.49 (dd, *J* =8.0, 7.6 Hz, 3H), 6.79 (d, *J* =16.0 Hz, 2H), 4.83 (2H, s, CH_2_), 2.56 (3H, s, CH_3_); ^13^C-NMR; *δ* =197.59 (CO, ketone), 165.81 (CO, ester), 165.69 (CO, amide), 143.96, 138.86, 137.35 135.20, 132.92, 130.22, 129.28, 129.02, 123.89, 118.51, 118.46, 118.28, 62.71 (CH_2_), 26.24 (CH_3_) (Figure S9); Molecular formula: C_19_H_16_ClNO_4_; Elemental Analysis: Calculated: (C, H) 63.78, 4.51; Found: (C, H) 63.76, 4.49.

*(10) (5e) 2-(3-Acetylphenylamino)-2-Oxoethyl 3-(4-Hydroxyphenyl) Propanoate*. Solid; Mp: 241-243°C; R_f_: 0.48, FT-IR (*ν*_max_, cm^−1^): 3432, 3265, 2934, 2849, 1733 (CO ester), 1651 (CO amide), 1596 (C=C aromatic); ^1^H-NMR; *δ* =10.32 (1H, s, NH), 9.19 (1H, s, OH), 8.16 (s, 1H), 7.82 (d, *J* =8.4 Hz, 1H), 7.66 (d, *J* =7.2 Hz, 1H), 7.45 (t, *J* =7.6 Hz, 1H), 7.02 (d, *J* =8.0 Hz, 2H), 6.66 (d, *J* =7.6 Hz, 2H), 4.67 (2H, s, CH_2_), 2.77 (t, *J* =6.8 Hz, 2H, CH_2_), 2.66 (t, *J* =7.2 Hz, 2H, CH_2_), 2.54 (3H, s, CH_3_): ^13^C-NMR; *δ* =197.61 (CO, ketone), 172.06 (CO, ester), 166.86 (CO, amide), 155.68, 138.85, 137.37, 130.47, 130.29, 129.15, 123.85, 123.67, 118.15, 115.16, 62.5 (CH_2_),35.35 (CH_2_), 29.40 (CH_2_), 26.75 (CH_3_) (Figure S10); Molecular formula: C_19_H_19_NO_5_; Elemental Analysis: Calculated: (C, H) 66.85, 5.61; Found: (C, H) 66.83, 5.59.

### 2.3. Mushroom Tyrosinase Inhibitory Activity

*In vitro* antityrosinase potential was determined following the method previously described with little modifications [33, 34]. Briefly, 140 *μ*L of sodium phosphate buffer, 20 *μ*L (30 U/mL) of the enzyme mushroom tyrosinase, and 20 *μ*L of the tested compound were added in a well of a 96-well microplate. After a preincubation of 10 minutes at room temperature of 20 *μ*L (0.85 mM), the substrate L-DOPA was added followed by an incubation of 20 minutes at the same temperature. Subsequently, the absorbance of the intermediate dopachrome was recorded by using an Opti Max tunable microplate reader (Sunnyvale, USA) at 472 nm. The standard inhibitors were kojic acid and arbutin. The negative inhibitor was a phosphate buffer without an inhibitor. All the compounds were dissolved in DMSO, and blank tests were performed with DMSO. IC_50_ values were calculated by Origin (8.6, 64-bit).

### 2.4. Mushroom Tyrosinase Inhibitory Kinetics

Based upon the antityrosinase assay, the most potent derivative (5c) was chosen for kinetic inhibition studies using enzyme and L-DOPA as a substrate [35–37]. Various concentrations of the inhibitor 5c were among 0.00 to 0.013 *μ*M and that of L-DOPA was 0.00 to 0.0064 *μ*M during all kinetic assays. For preincubation and calculations, the same protocol was followed as described in *in vitro* tyrosinase inhibition assay and continuous monitoring at 30 s interval for 5 minutes after adding the enzyme tyrosinase, and the formation of dopachrome was measured at 475 nm. The enzyme inhibitory action and the kind of inhibition were determined by the Lineweaver-Burk and Dixon plots. 1/V (inverse of velocities) versus 1/[S] (inverse of substrate concentration) mM^−1^ were plotted to determine inhibition constant *K*_*i*_.

### 2.5. Computational Studies

#### 2.5.1. Ligand Preparation

ChemDraw Professional 15.2 was used to sketch all the ligands and LigPrep (Schrödinger) to prepare in their neutral form. The conformation of the prepared ligands was optimized in the OPLS-3 force field for further docking analysis.

#### 2.5.2. Retrieval of Mushroom Tyrosinase in Maestro

For this purpose, the crystal structure of target protein (PDB ID 2Y9X) was retrieved from RCSB and prepared in “Protein Preparation Wizard” workflow in Maestro Schrödinger for adding hydrogens, adjusting protonation and bond orders appropriate for pH 7. The water molecules beyond 5 Å from het groups were removed and minimized the prepared protein structure for further grid generation and docking analysis.

#### 2.5.3. Receptor Grid Generation and Docking

For grid generation, the catalytic pocket is selected from its cocrystallized ligand and literature [38]. The grid was generated by specifying the cocrystallized ligand tropolone of the active site of the target protein. The receptor grid box was defined as 20 Å box. After grid preparation, Glide_dock_XP precision docking experiment was performed with default docking setup parameters reporting the 15 top-ranked poses per ligand [39]. The predicted binding scores (binding energies) and proper orientation of ligands within catalytic region of tyrosinase was also performed. Finally, the most favorable binding mode of active compounds within the binding pocket was investigated in terms of docking score and 3D graphical images of the binding pose of the best docked score was also done with Maestro (Schrödinger).

## 3. Results and Discussions

### 3.1. In Vitro Mushroom Tyrosinase Inhibition Assay

**Figure 1:**
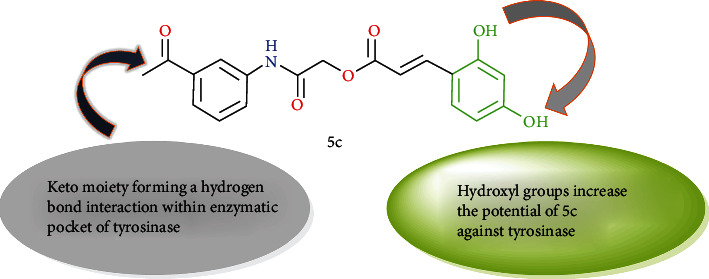
Structural components of representative compound 2-(3-acetylphenylamino)-2-oxoethyl(E)-3-(2,4-dihydroxyphenyl)acrylate (5c) responsible for its enhanced tyrosinase inhibition.

Benzoic and cinnamic acid analogues 3(a–e) and 5(a–e) were designed and synthesized to evaluate *in vitro* tyrosinase inhibition activity.  Table 1 demonstrates that title amide derivatives bearing a cinnamic acid moiety showed better tyrosinase inhibition than reference drugs kojic acid and arbutin and benzoic acid 3(a–e) analogues. The cinnamic acid analogues 5a, 5b, 5c, and 5d have different substitutions on the phenyl ring. The analogue 5a is an unsubstituted cinnamic acid analogue, and 5b and 5c contain 4-hydroxyl and 2,4-dihydroxyl substituted cinnamic acid moieties, respectively, whereas the inhibitor 5d bears a chloro substituent at the *para* position. The hydroxyl substitution at the second and fourth positions of the cinnamic acid phenyl ring in 5c resulted in a considerable rise in the enzyme inhibitory activity (Table 1). The phenolic hydroxyls play a crucial role in tyrosinase inhibition as the natural substrates of tyrosinase, L-tyrosine, and L-DOPA also bear the phenolic hydroxyls [40]. A naturally occurring cinnamic acid has been reported with extensive physiological activities including tyrosinase inhibitory action [41]. The compounds 5a (IC_50_ 46.43 *μ*M), 5b (IC_50_ 309.20 *μ*M), and 5d (IC_50_ 61.62 *μ*M) displayed lower inhibitory activity than 5c (IC_50_ 0.0020 *μ*M) compared to reference inhibitor kojic acid (IC_50_ 16.69 *μ*M) and arbutin (IC_50_ 191.17 *μ*M). It is reported here that the *ortho-* and *parahydroxyls* of the phenyl ring in 5c appear to show an increased tyrosinase inhibitory activity (Figure 1).

### 3.2. Enzyme Inhibitory Kinetics

**Figure 2:**
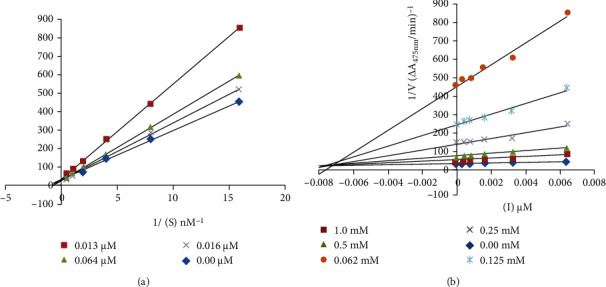
(a) Lineweaver-Burk and (b) Dixon plots for the diphenolase inhibitory activity of mushroom tyrosinase by compound 2-(3-acetylphenylamino)-2-oxoethyl(E)-3-(2,4-dihydroxyphenyl)acrylate (5c) in the presence of different concentrations of L-DOPA (L-3,4-dihydroxyphenylalanine).

The enzyme inhibitory interaction mechanism of 3(a–e) and 5(a–e) with the binding site of mushroom tyrosinase was determined using Michaelis-Menten kinetic studies. The inhibitors exhibit a dose-dependent inhibition of the enzyme tyrosinase. A striking reduction in the reaction rate in the presence of inhibitors refers to a decrease in the final absorbance in comparison to control without inhibitors. The inhibitory index of all these tested compounds varied depending upon the position of various substituents and classes of compounds [27]. Inhibition kinetics was analyzed by the Lineweaver-Burk and Dixon plots. The kinetics analyses of 1/V_max_ vs. 1/[S] at different doses of 5c reveal that the Michaelis-Menten constant (*K*_*m*_) changes while that of 1/V_max_ remained the same representing the competitive nature of the most potent inhibitor (5c) (Figure 2(a)). The dissociation constant *K*_*i*_ for 5c was 0.0072 *μ*M calculated by Dixon plots as shown in Figure 2(b) [27, 42].

#### 3.2.1. Inhibitory Action of Compound 2-(3-Acetylphenylamino)-2-Oxoethyl (E)-3-(2,4-Dihydroxyphenyl) Acrylate (5c) Using L-DOPA as Substrate

**Figure 3:**
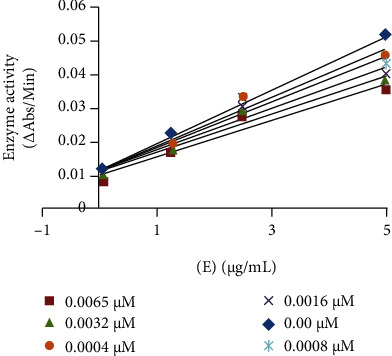
Catalytic action of mushroom tyrosinase and compound 5c.

The plot of the remaining enzyme activity at different concentrations (0.00, 1.25, 2.5, and 5 *μ*g/mL) of enzyme vs. various doses (0.00, 0.0004, 0.0008, 0.0016, 0.0032, 0.0065 *μ*M) of the inhibitor 5c for L-DOPA catalysis showed a group of straight lines with varying slopes and intersecting on the same point on the *y*-axis preferring the reversible effect of 5c on tyrosinase (Figure 3). The results suggested that 5c effectively inhibits the enzyme tyrosinase by binding to its active site reversibly [35].

### 3.3. In Silico Studies

#### 3.3.1. Binding Pocket Analysis of Analogues 3(a-e) and 5(a-e) against Mushroom Tyrosinase

**Figure 4:**
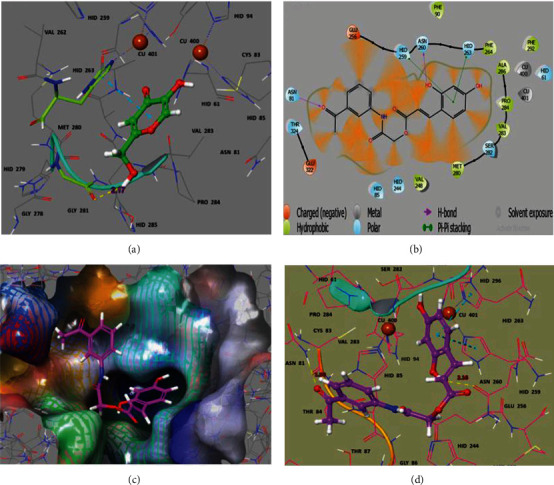
Ball and stick illustration of the docking pose of (a) Kojic acid (green), (b) 2D ligand interaction diagram, (c) surface and stick representation (purple), and (d) ball and stick representation of 2-(3-acetylphenylamino)-2-oxoethyl(E)-3-(2,4-dihydroxyphenyl)acrylate (5c), (blue) in the binding pocket of tyrosinase 2Y9X. The 5c is binding closer to Cu^2+^(brown) in the active site.

A computational molecular docking study was also conducted to examine the binding conformations of all the synthesized compounds within the catalytic pocket of enzyme tyrosinase. The docked ligand-protein complexes were investigated based on docking score (kcal/mol) and the hydrophobic/philic bonding interaction pattern. The docking scores had little fluctuations, and the comparison depicted no significant energy difference among all docked molecules due to similar basic skeleton of the ligands. Therefore, majority of the ligands showed efficient docking energy values. From docking results and *in vitro* enzyme inhibitory assay, the most active compound 5c (-6.568 kcal/mol) was visualized to determine its interactions in the catalytic site of the protein tyrosinase (Figure 4). Two strong hydrogen bond contacts were observed. The orthohydroxyl phenolic moiety of 5c is picking up a hydrogen bond (2.16 Å) interaction with the side chain carbonyl of Asn 260, and this phenolic ring is further stabilized by *π*-*π* stacking with side chain His 259 and His 263. The keto carbonyl oxygen from the tail moiety of this compound is interacting through another hydrogen bond with neighboring Asn 81 (1.90 Å) predicting a competitive type of inhibition for 5c. In the tyrosinase signature, His 61, His 85, His 94 (Cu 400), His 259, His 263, and His 296 (Cu 401) are conserved residues in the core region. Previous studies exposed that Asn 81, Asn 260, His 259, His 263, and Met 280 are the crucial residues responsible for stabilizing the protein inhibitor complex [43].

#### 3.3.2. Chemoinformatics and Lipinski's Rule of Five (RO5)

The Molsoft tool used to predict pharmacokinetics properties such as the number of hydrogen bond acceptor/donor, solubility, polar surface area, and molar volume. The standard range for molar molecular weight (160-500), polarizability ≤ 5, and solubility ≤ 4 has been reported in literature [44]. The computational results predicted that the molecular weight of 5c is 355.11 g/mol, and it possesses 6 HBA (≤10), 3 HBD (≤5), 2.72 LogP (≤5), -3.11 mol/L LogS, and 91.64 A^2^ PSA, and the molar volume of 355.34 A^3^ proved to have a drug-like behavior within the standard range (Table 2). Furthermore, Lipinski's rule of five (RO5) states nothing about structural features found in drugs and nondrugs for their medicinal potential.

## 4. Conclusions

The findings of the current study have provided a comprehensive overview of tyrosinase inhibitory activity of acetophenone analogues. According to the results presented, cinnamic acid derivatives 5(a–e) are more potent than benzoic acids 3(a–e), likely due to the additional *α*,*β*-unsaturation present in the 5(a–e). Among all the synthesized compounds, 2-(3-acetyl phenylamino)-2-oxoethyl(E)-3-(2,4-dihydroxy phenyl)acrylate (5c) exhibited the most promising inhibitory activity against tyrosinase (IC_50_ 0.0020 *μ*M) better than the standard kojic acid (IC_50_ 16.69 *μ*M) and arbutin (IC_50_ 191.17 *μ*M). Moreover, in the cinnamate amide 5(a–e) scaffold, the presence of -OH groups is of great interest for antityrosinase activity. In addition, from benzoic acid analogues, 3c (2-((3-Acetylphenylamino)-2-oxoethyl 2,4-dihydroxy benzoate) also displayed considerable activity against tyrosinase (IC_50_ 27.35 *μ*M) and has potential to be explored further for antityrosinase and cosmetic drug discovery and its clinical exploitation.

## Figures and Tables

**Table 1:** The yield, substitution pattern, mushroom tyrosinase inhibition and docking scores of analogues 3(a–e) and 5(a–e).

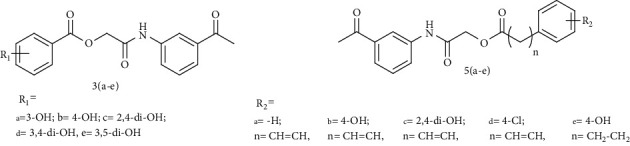					
Compounds	Yield (%)	Substitution pattern		Mushroom tyrosinase inhibition IC_50_ ± SEM (*μ*M)	Docking score (kcal/mol), PDB ID 2Y9X
		*R*_1_	*R*_2_		
3a	81	3-OH		322.68 ± 16.9	-2.362
3b	78	4-OH		287.53 ± 32.2	-4.295
3c	74	2,4-di-OH		27.35 ± 3.6	-5.564
3d	69	3,4-di-OH		127.65 ± 8.5	-4.569
3e	76	3,5-di-OH		255.31 ± 14.2	-3.984
5a	75		-H	46.43 ± 5.8	-6.173
5b	73		4-OH	309.20 ± 36.8	-4.264
5c	71		2,4-di-OH	0.0020 ± 0.0002	-6.568
5d	78		4-cl	61.62 ± 11.4	-5.299
5e	72		4-OH	N.d.^∗^	-4.628
Arbutin				191.17 ± 5.5	-4.759
Kojic acid				16.69 ± 2.8	-3.792

3a: 2-(3-acetylphenylamino)-2-oxoethyl-3-hydroxybenzoate; 3b: 2-(3-acetylphenylamino)-2-oxoethyl-4-hydroxybenzoate; 3c: 2-(3-acetylphenylamino)-2-oxoethyl 2,4-dihydroxybenzoate; 3d: 2-(3-acetylphenylamino)-2-oxoethyl 3,4-dihydroxybenzoate; 3e: 2-(3-acetylphenylamino)-2-oxoethyl 3,5-dihydroxybenzoate; 5a: 2-(3-acetylphenylamino)-2-oxoethylcinnamate; 5b: 2-(3-acetylphenylamino)-2-oxoethyl(E)-3-(4-hydroxyphenyl) acrylate; 5c: 2-(3-acetylphenylamino)-2-oxoethyl(E)-3-(2,4-dihydroxyphenyl)acrylate; 5d: 2-(3-acetylphenyl amino)-2-oxoethyl(E)-3-(4-chlorophenyl)acrylate; 5e: 2-(3-acetylphenylamino)-2-oxoethyl3-(4-hydroxyphenyl) propanoate; SEM: the standard error mean; N.d.^∗^: not determined.

**Table 2:** Pharmacokinetic assessment of synthesized drugs 3(a–e) and 5(a–e).

Compound	M.Wt ≤ 500	HBA ≤ 10	HBD ≤ 5	LogP ≤ 5	LogS (mol/L) ≤ −4	PSA ≤ 140 (A^2^)	Vol (A^3^)	Drug likeness score > 0	RO5
3a	313.10	5	2	2.54	-2.65	75.69	301.07	0.16	Yes
3b	313.10	5	2	2.51	-2.78	75.69	300.99	0.49	Yes
3c	329.09	6	3	2.76	-3.23	92.24	312.25	0.74	Yes
3d	329.09	6	3	1.96	-2.40	91.17	313.72	0.60	Yes
3e	329.09	6	3	2.10	-2.62	93.31	311.77	-0.08	Yes
5a	323.12	4	1	3.52	-3.67	57.48	334.19	-0.60	Yes
5b	339.11	5	2	2.96	-3.33	75.09	344.74	-0.05	Yes
5c	355.11	6	3	2.72	-3.11	91.64	355.34	-0.08	Yes
5d	357.08	4	1	4.12	-4.73	57.48	351.39	-0.04	Yes
5e	341.13	5	2	2.82	-3.30	75.09	336.60	-0.01	Yes
Kojic acid	142.03	4	2	-1.07	-0.08	56.19	148.32	-1.04	Yes
Arbutin	272.09	7	5	-1.14	-0.96	97.71	228.69	-1.04	Yes

M.Wt: molecular weight; HBA: number of hydrogen bond acceptor; HBD: number of hydrogen bond donor, LogP: partition coefficient; LogS: lipophilicity of water; PSA: total polar surface area; RO5: Lipinski's rule of 5.

## Data Availability

Data could be provided upon request from the shared corresponding author Dr. Zaman Ashraf, mzchem@yahoo.com
