# Supplementary material for: Molecular Docking, Synthesis, and Tyrosinase Inhibition Activity of Acetophenone Amide: Potential Inhibitor of Melanogenesis
Source: Biomed Res Int. 2022 Jan 11;2022:1040693. doi: 10.1155/2022/1040693 (PMC8766184; doi:10.1155/2022/1040693)
Supplement: Supplementary Materials — Figures S1-S10 are available online as supplementary materials. [file 1040693.f1.docx]

**Spectral data:**

**Note:** All signals coming from solvents residue (ethyl acetate, acetone etc) are not integrated.

**Compound 3a:**

1. **^1^H NMR**


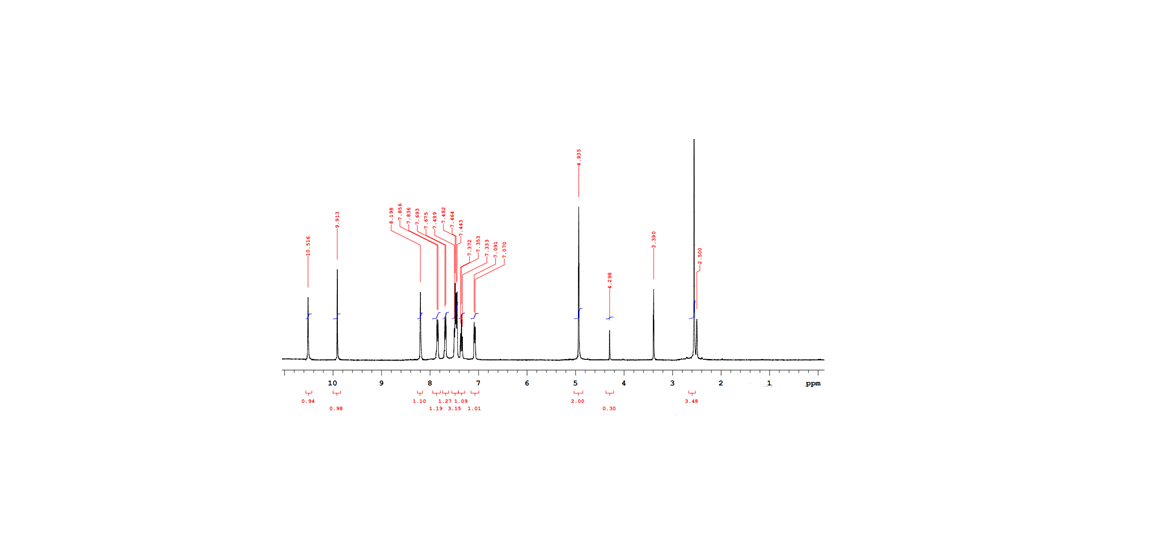


1. **^13^C NMR**


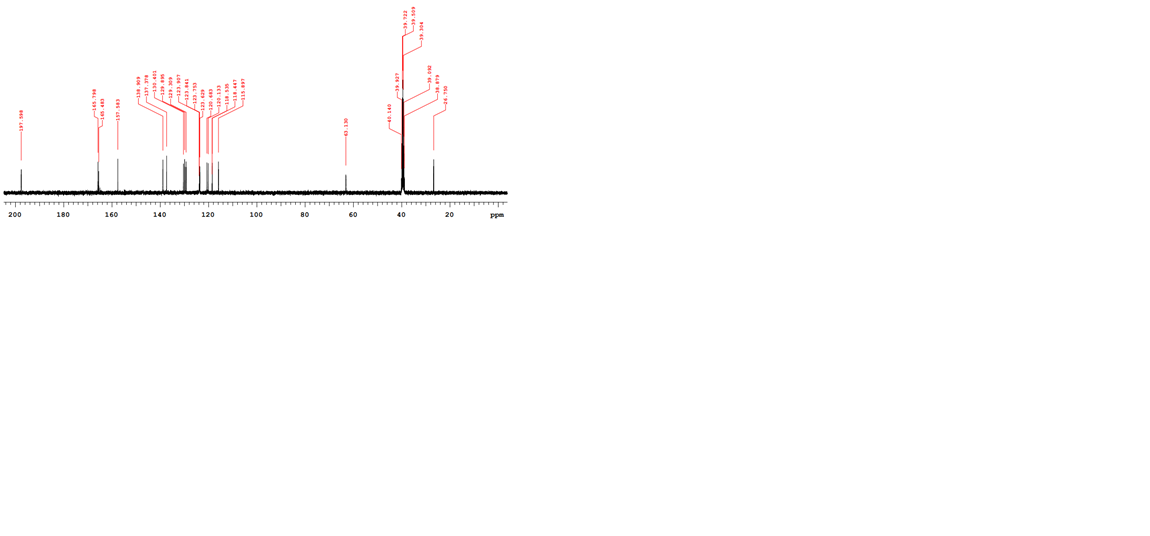


Figure S1. ^1^H, ^13^C NMR spectra of compound 3a

**Compound 3b:**

1. **^1^H NMR**


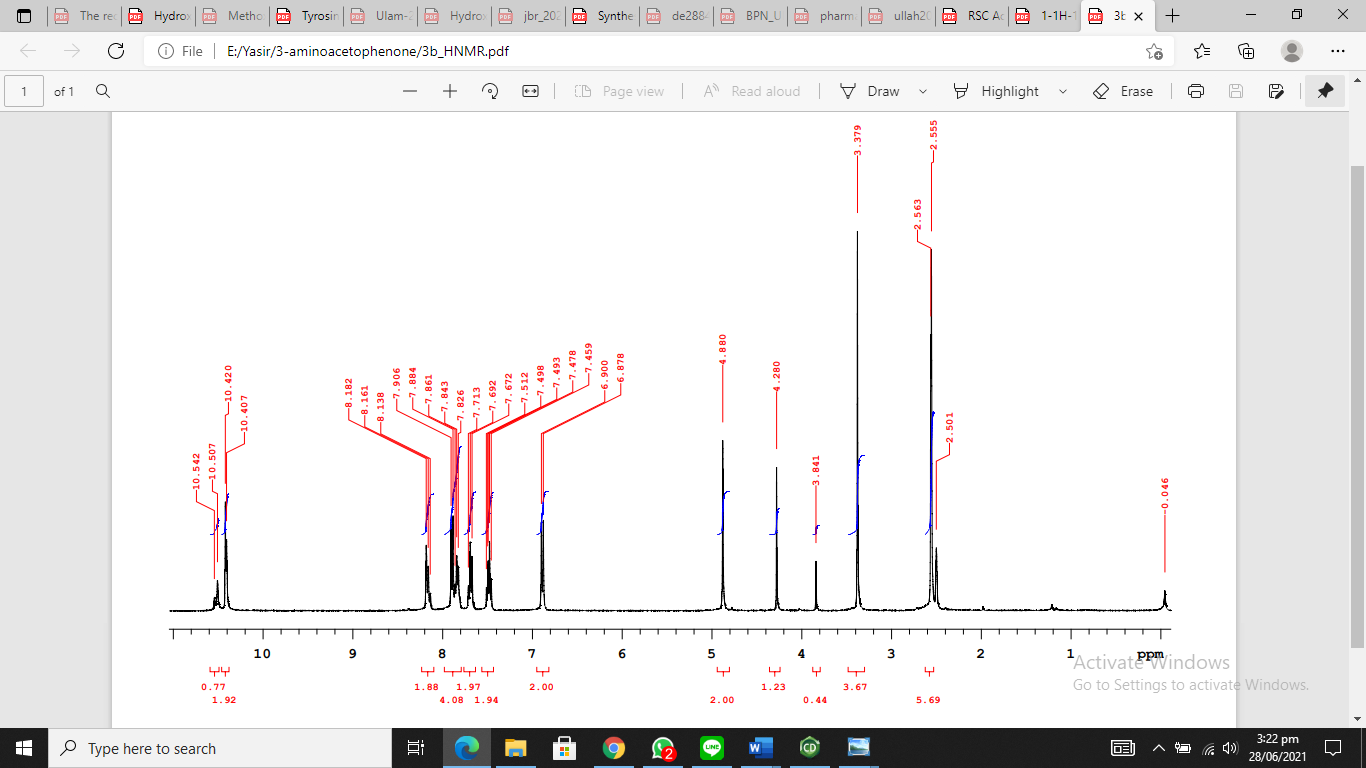


1. **^13^C NMR**


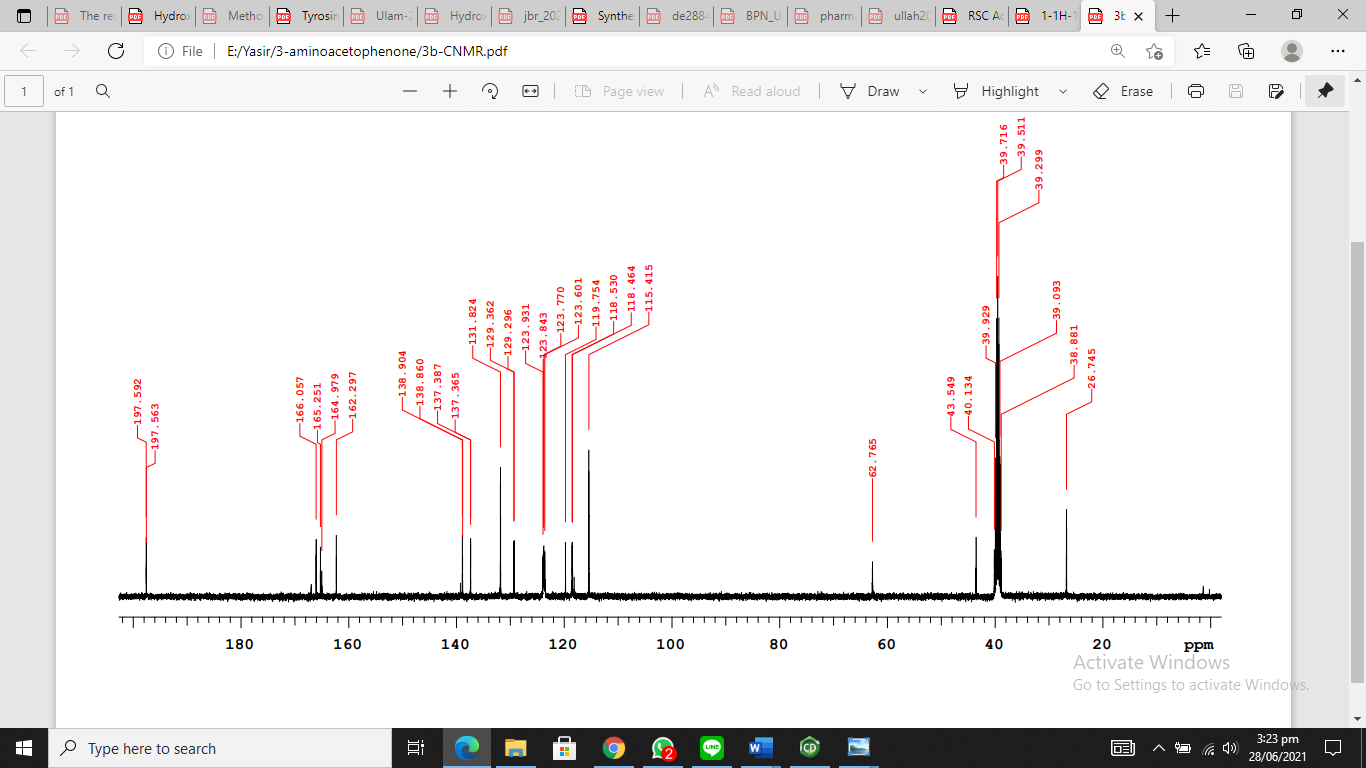


Figure S2. ^1^H, ^13^C NMR spectra of compound 3b

**Compound 3c:**

1. **^1^H NMR**


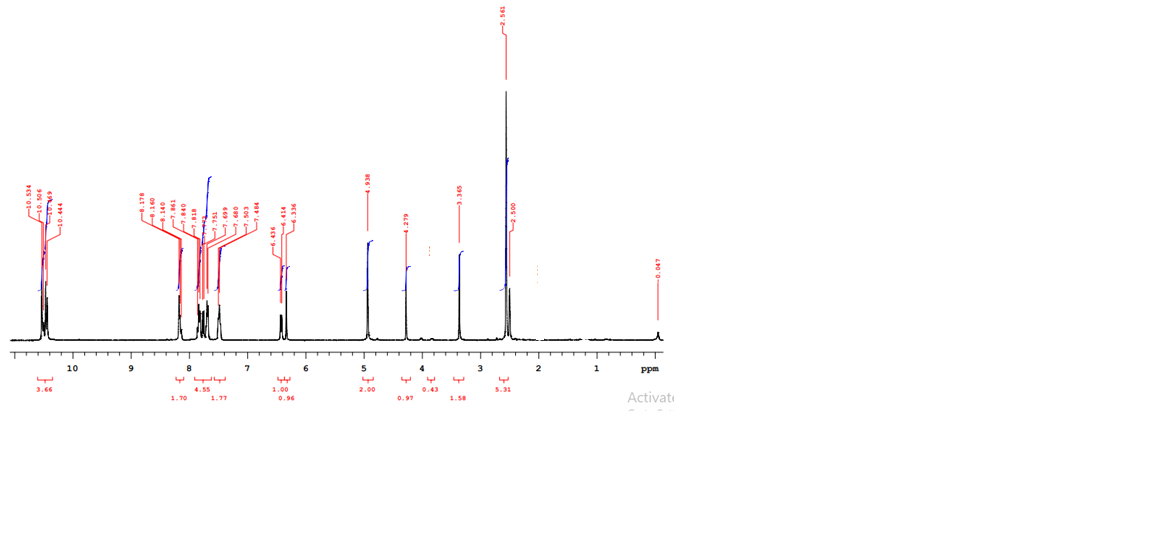


1. **^13^C NMR**


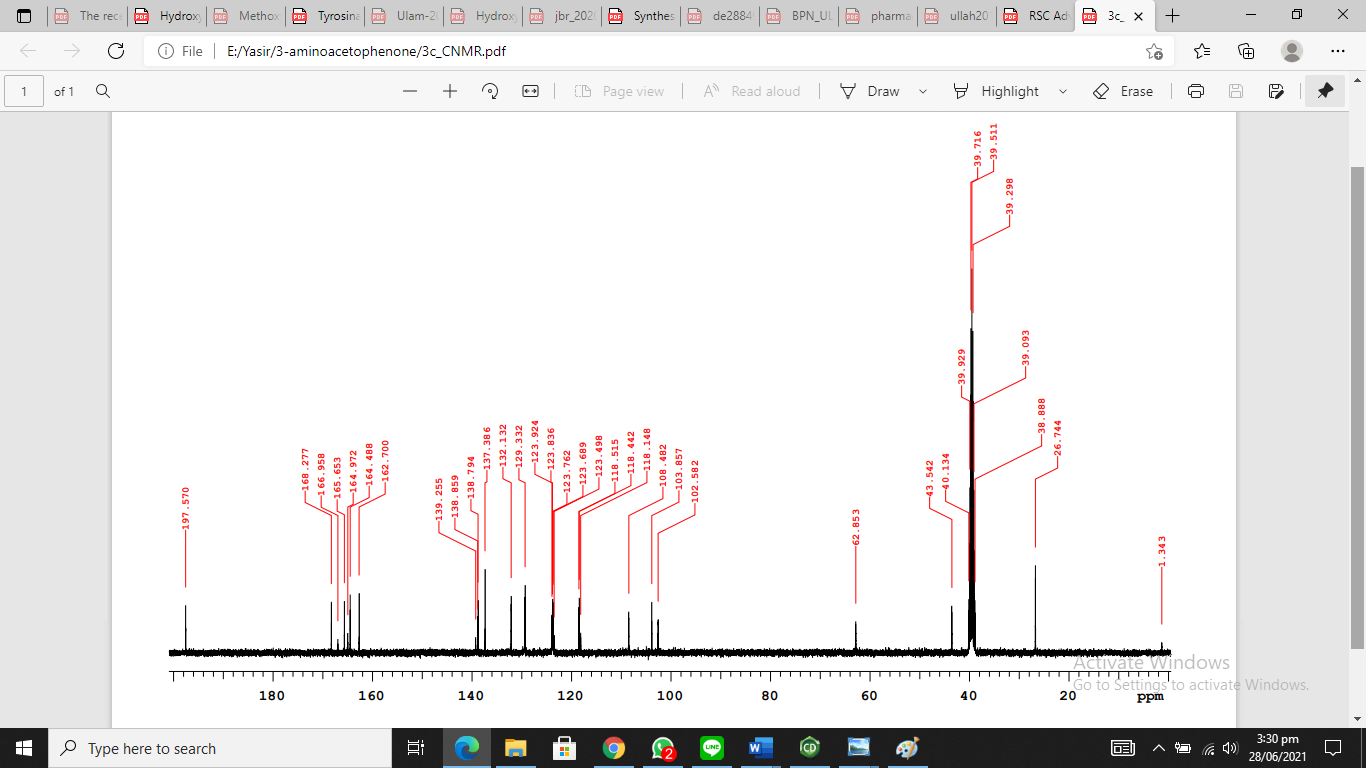


Figure S3. ^1^H, ^13^C NMR spectra of compound 3c

**Compound 3d:**

1. **^1^H NMR**


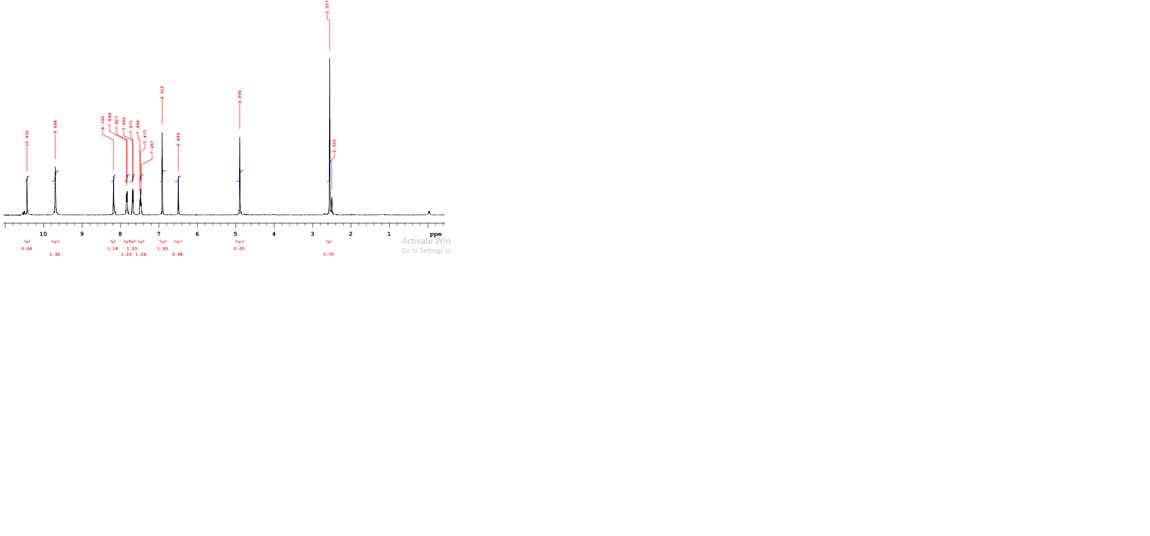


1. **^13^C NMR**


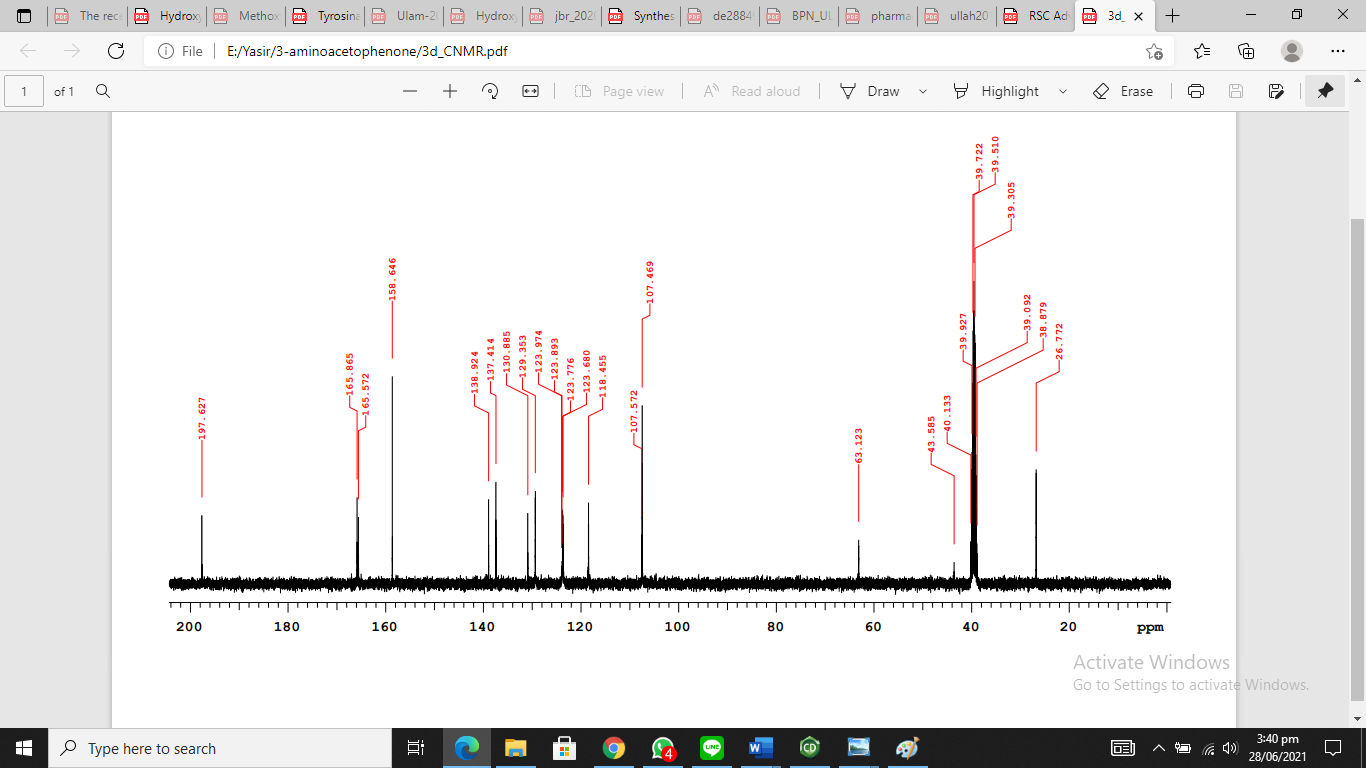


Figure S4. ^1^H, ^13^C NMR spectra of compound 3d

**Compound 3e:**

1. **^1^H NMR**


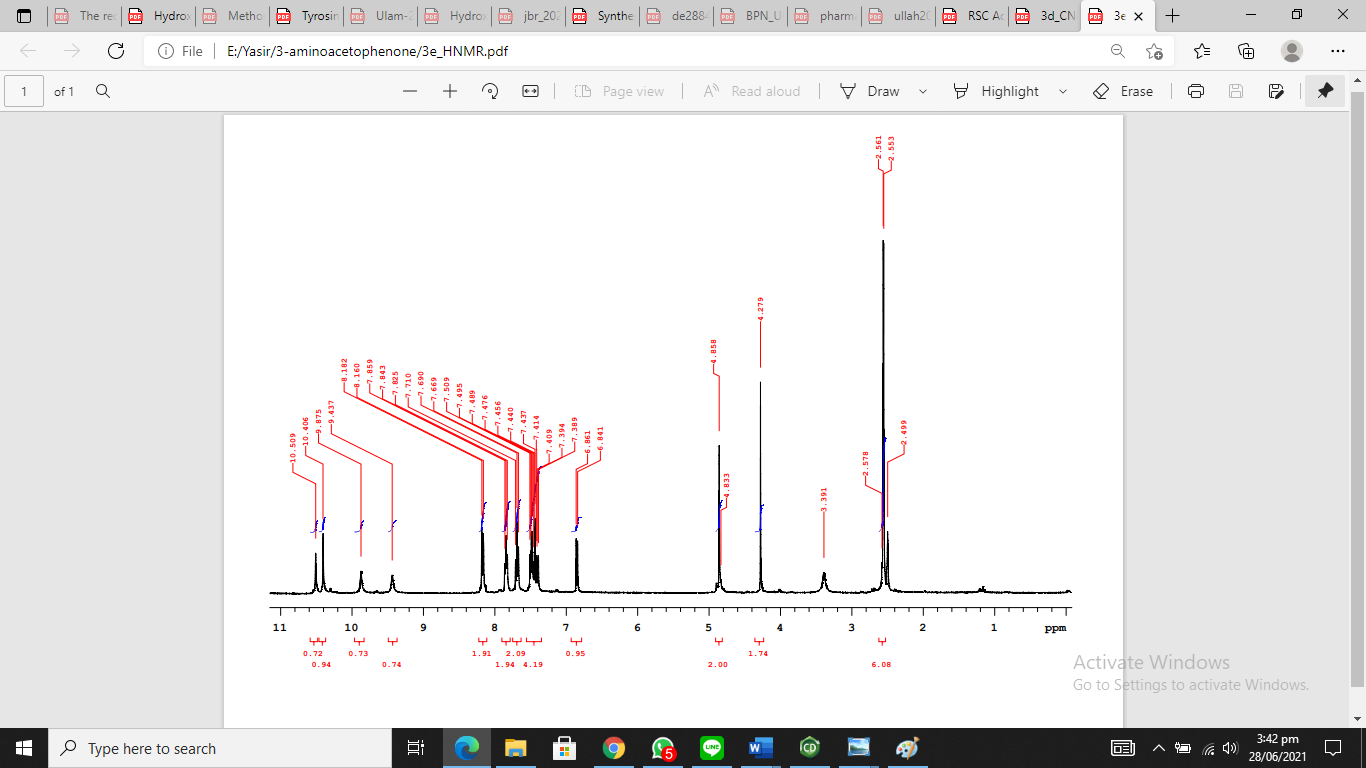


1. **^13^C NMR**


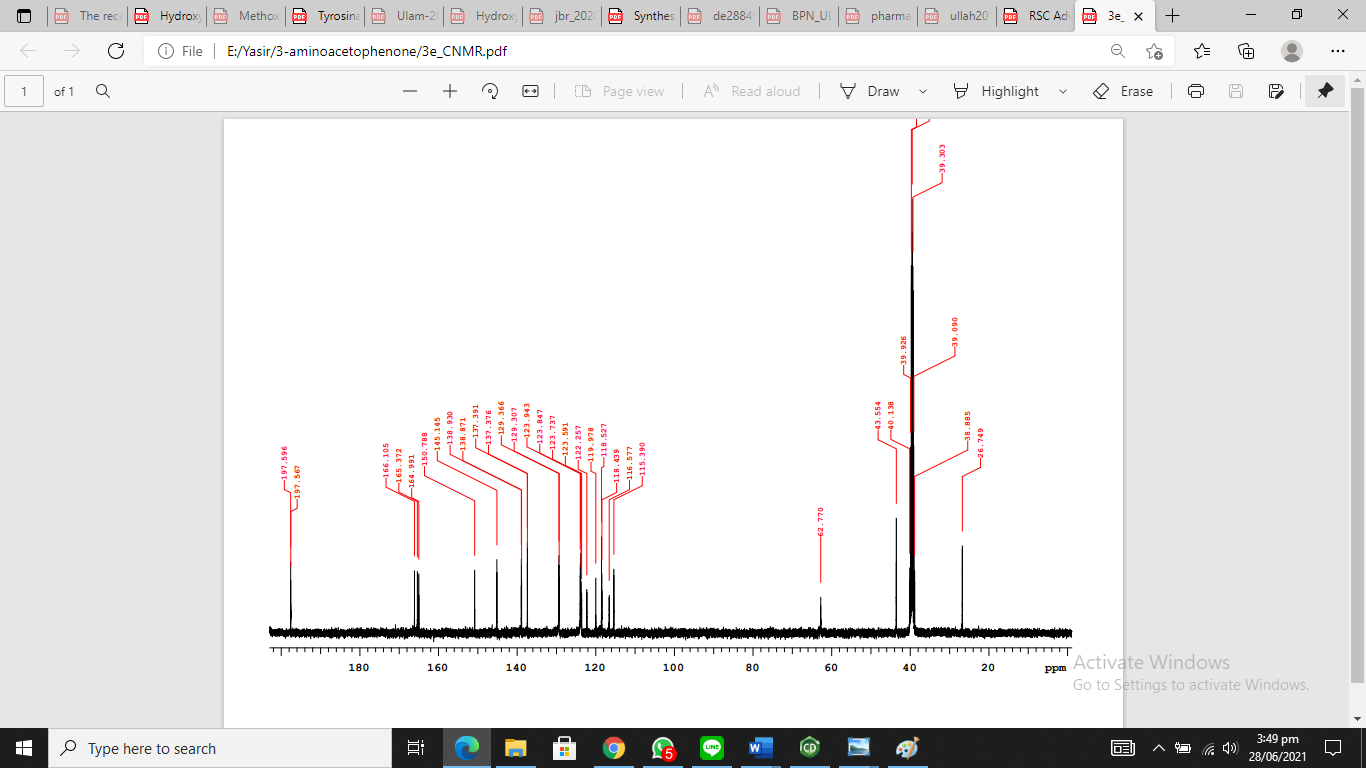


Figure S5. ^1^H, ^13^C NMR spectra of compound 3e

**Compound 5a:**

1. **^1^H NMR**


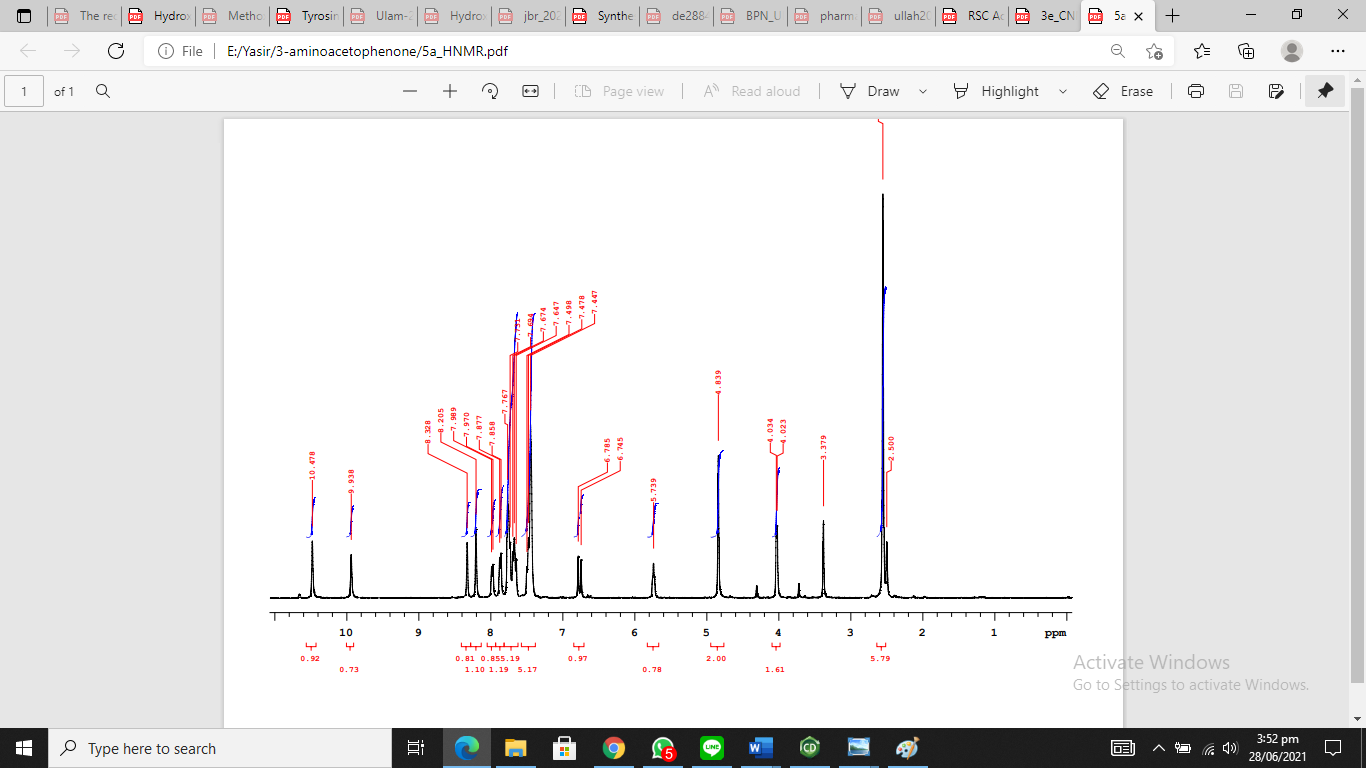


1. **^13^C NMR**


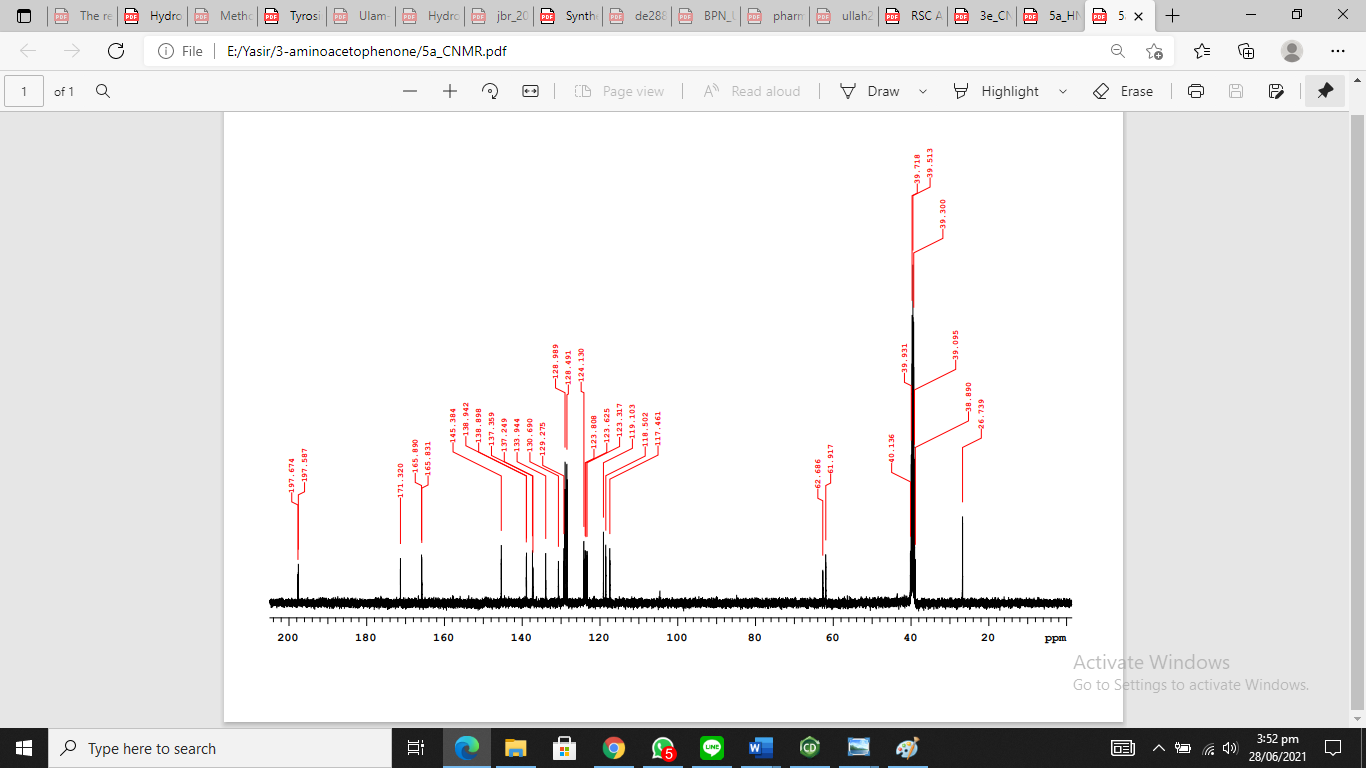


Figure S6. ^1^H, ^13^C NMR spectra of compound 5a

**Compound 5b:**

1. **^1^H NMR**


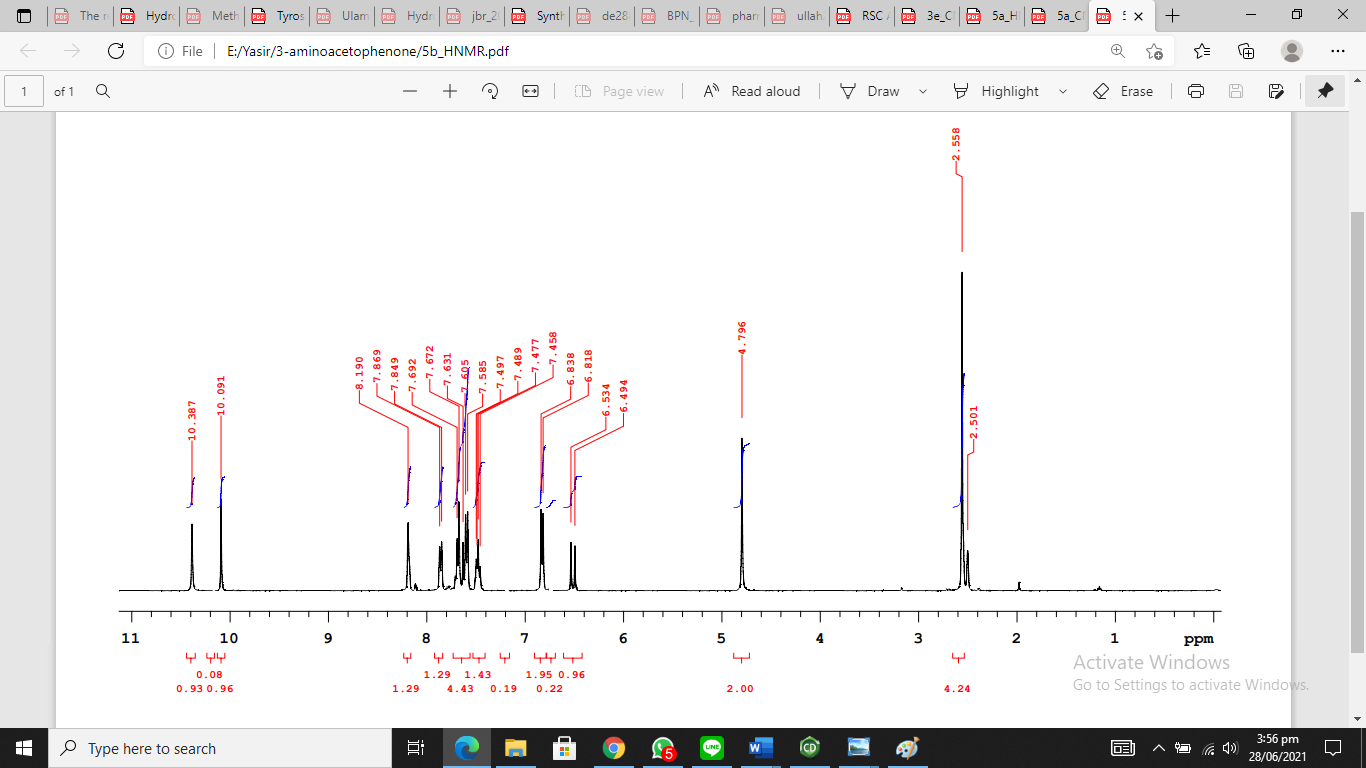


1. **^13^C NMR**


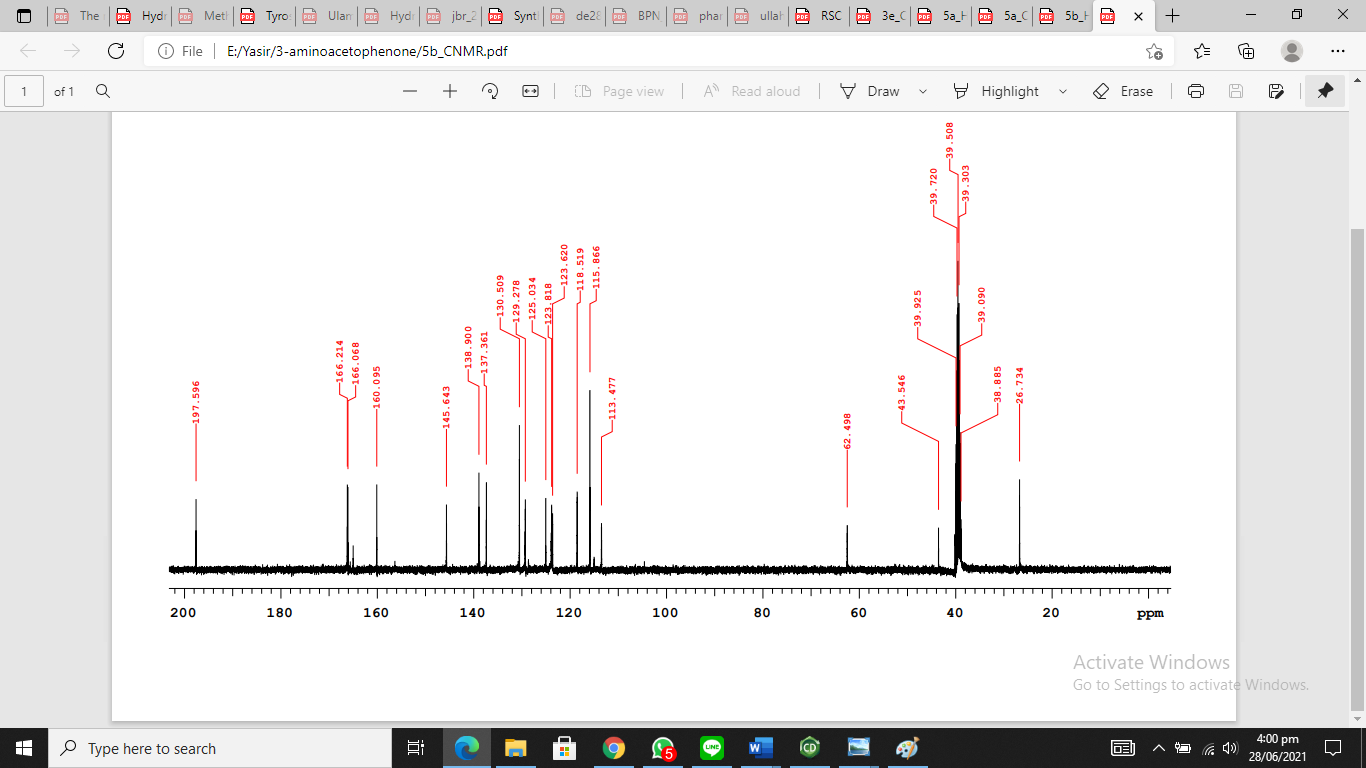


Figure S7. ^1^H, ^13^C NMR spectra of compound 5b

**Compound 5c:**

1. **^1^H NMR**


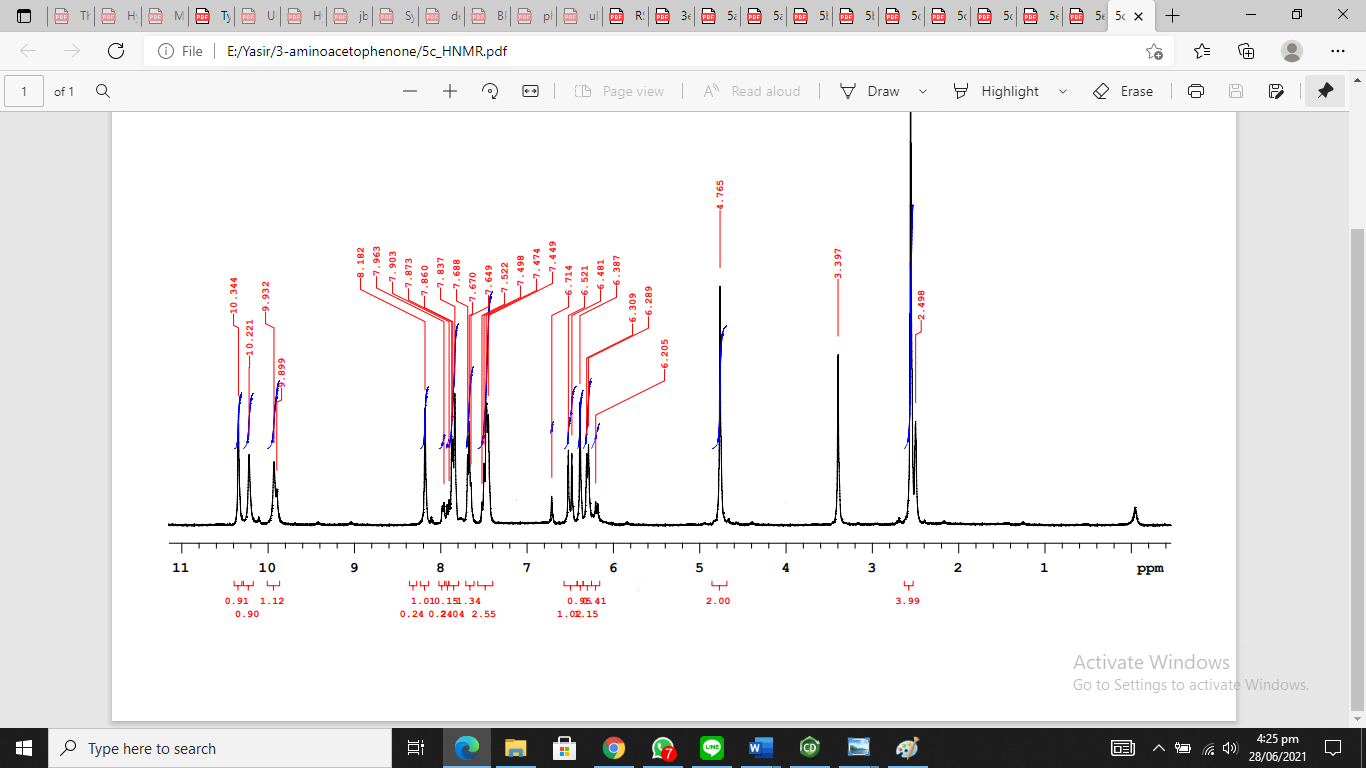


1. **^13^C NMR**


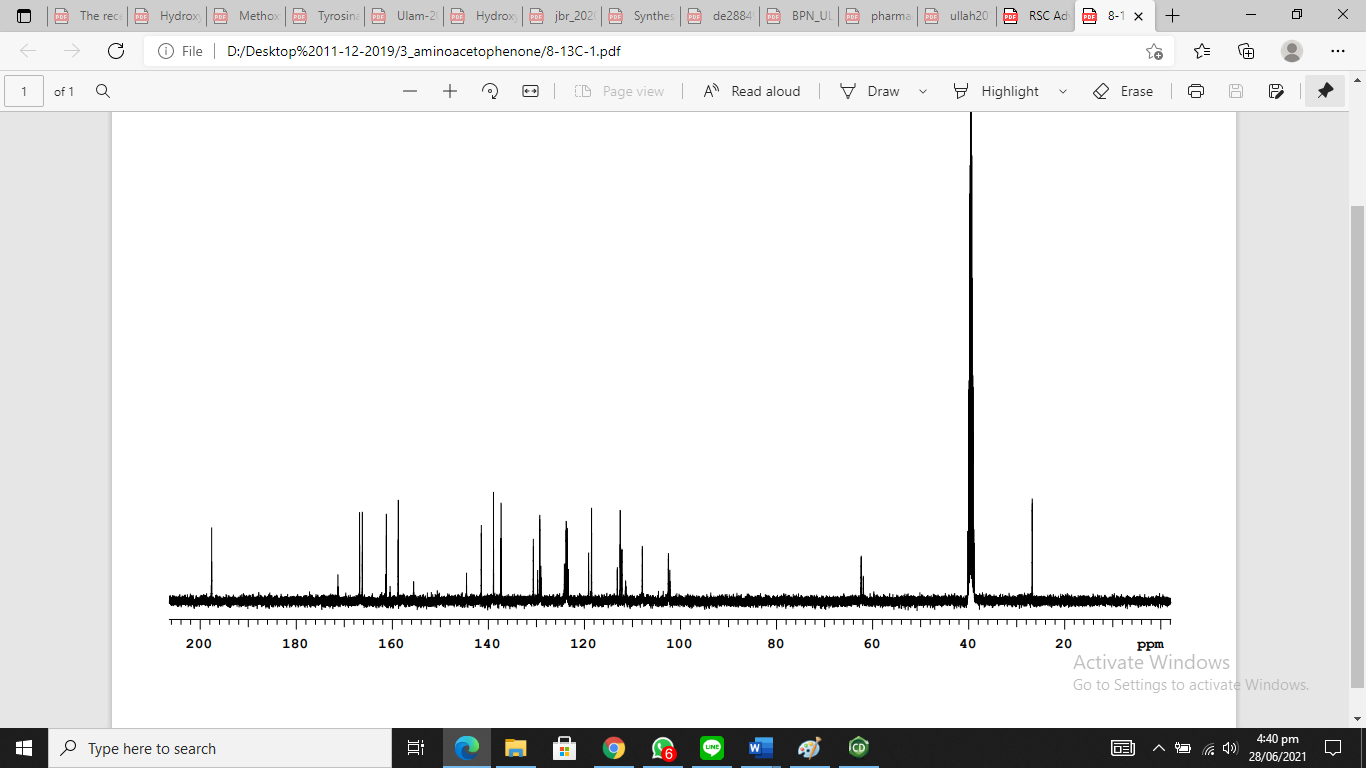


Figure S8. ^1^H NMR spectra of compound 5c

**Compound 5d:**

1. **^1^H NMR**


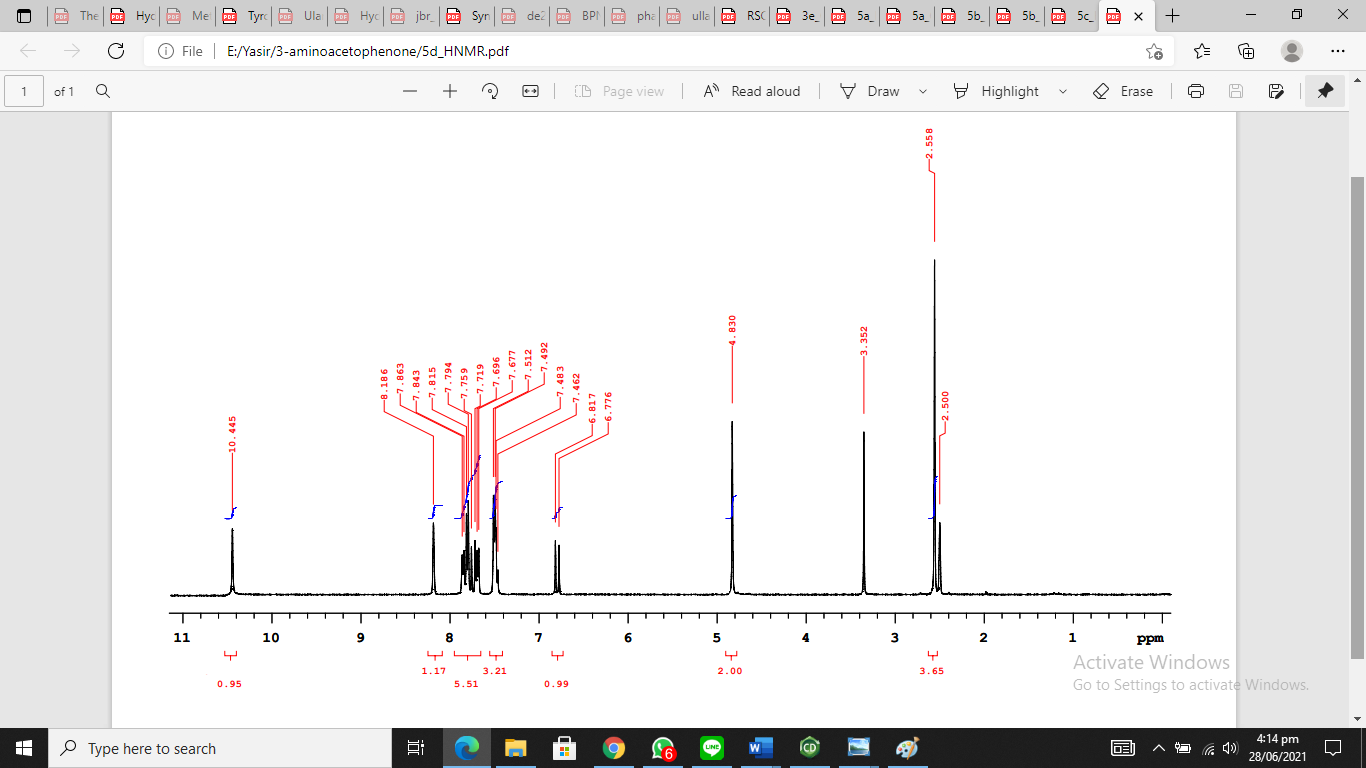


1. **^13^C NMR**


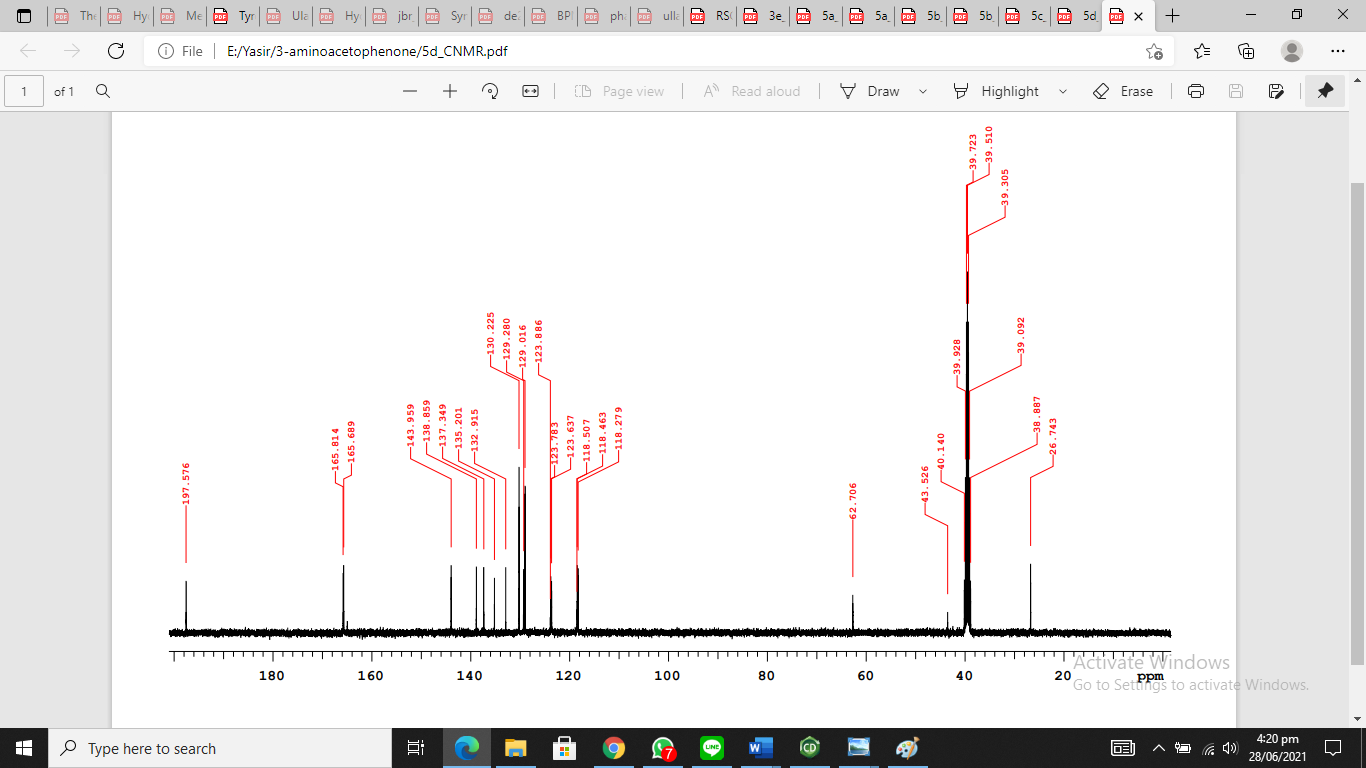


Figure S9. ^1^H, ^13^C NMR spectra of compound 5d

**Compound 5e:**

1. **^1^H NMR**


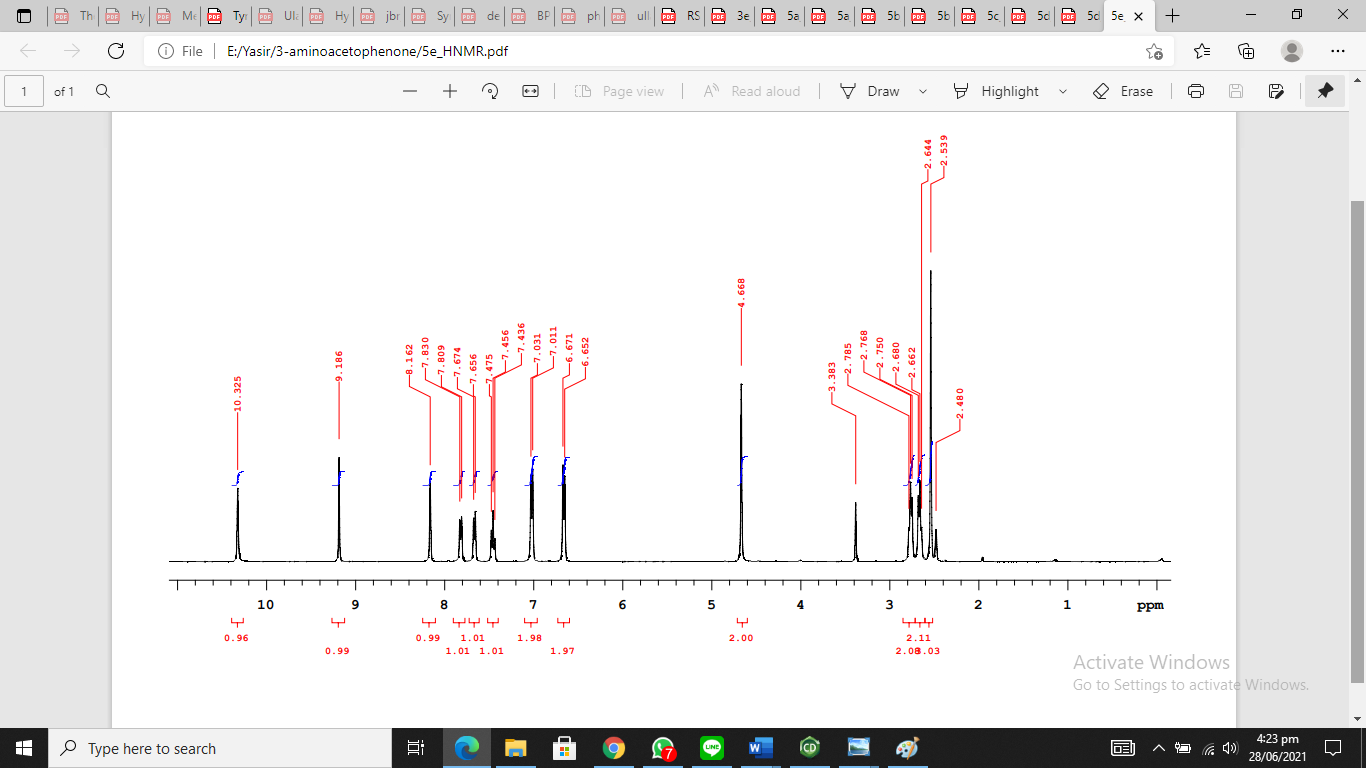


1. **^13^C NMR**


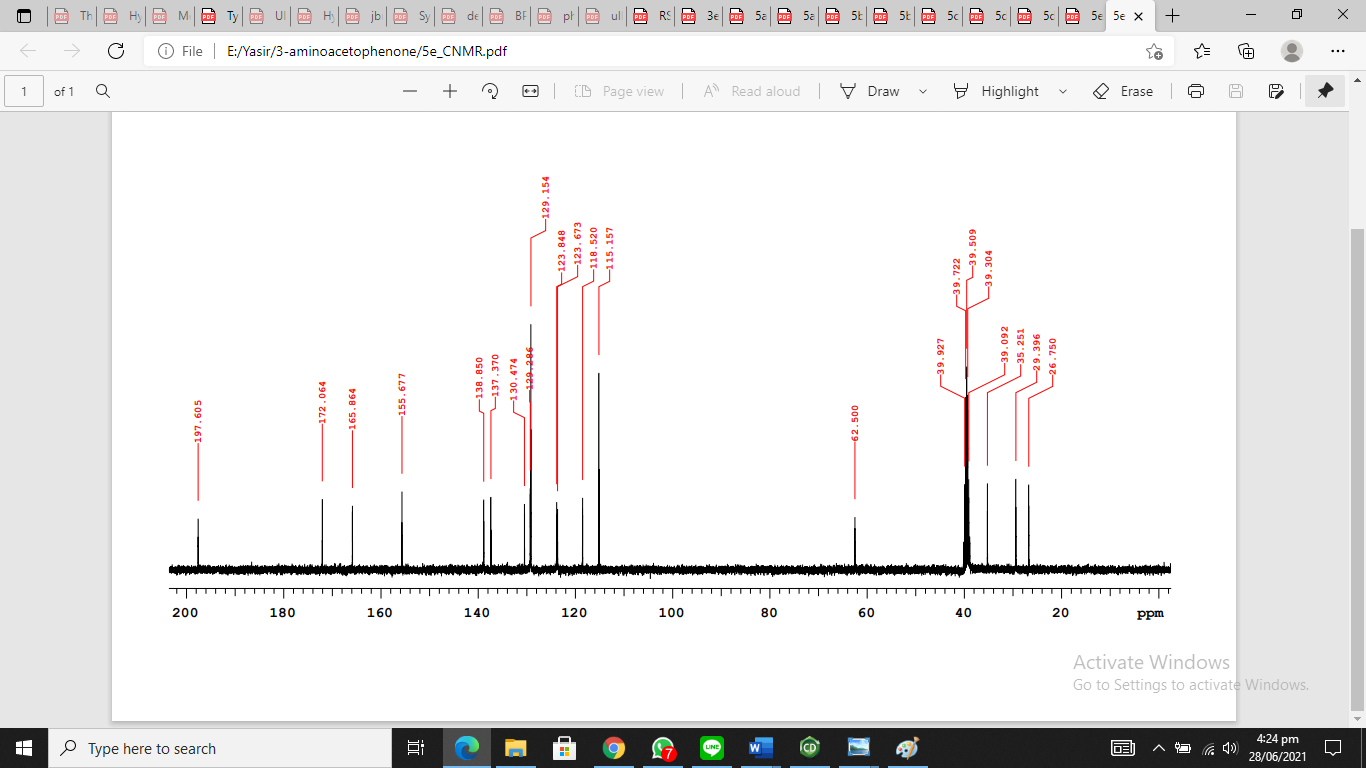


Figure S10. ^1^H, ^13^C NMR spectra of compound 5e
